# Deciphering Surface-Localized Structure of Nanodiamonds

**DOI:** 10.3390/nano14242024

**Published:** 2024-12-16

**Authors:** Li Ma, Zhijie He, Keyuan Chen, Hanqing Li, Yongzhi Wu, Jueyi Ye, Hongying Hou, Ju Rong, Xiaohua Yu

**Affiliations:** 1 Faculty of Materials Science and Engineering, Kunming University of Science and Technology, Kunming 650093, China; lima_zt@163.com (L.M.); 18541243341@163.com (Z.H.); cky_1999@163.com (K.C.); lhq202309@163.com (H.L.); yongz_wu@163.com (Y.W.); jueyi_y@163.com (J.Y.); hongyinghou@kust.edu.cn (H.H.); 2 Yunnan Key Laboratory of Integrated Computational Materials Engineering for Advanced Light Metals, Kunming 650093, China

**Keywords:** nanodiamonds, size effect, surface structure, core–shell structure, density functional theory, molecular dynamics simulation

## Abstract

Nanomaterials, heralded as the “new materials of the 21st century” for their remarkable physical and chemical properties and broad application potential, have attracted substantial attention in recent years. Among these materials, which challenge traditional physical boundaries, nanodiamonds (NDs) are widely applied across diverse industries due to their exceptional surface multifunctionality and chemical stability. Nevertheless, atomic-level manipulation of NDs presents considerable challenges, which require detailed structural analysis to thoroughly elucidate their properties. This study utilizes density functional theory (DFT), lattice dynamics, and molecular dynamics (MD) simulations to analyze the structural and property characteristics of NDs. Fine structural analysis reveals that, despite variations in particle size, surface layer thickness remains relatively constant at approximately 3 Å. DFT methods enable computation of the surface layer to capture subtle electronic characteristics, while the internal core is analyzed via MD. Further investigation into amorphous structure control indicates that ND surface amorphous structures with a packing coefficient above 0.38 are thermodynamically stable. This study offers a novel approach to nanomaterial control in practical applications by elucidating the core–shell interactions and surface structures of NDs.

## 1. Introduction

Nanotechnology, as a frontier science exploring the fundamental units of matter, is progressively uncovering the mysteries of the microscopic world [1]. At present, nanotechnology has already catalyzed a series of breakthroughs in the field of materials science, significantly driving industry transformation and demonstrating disruptive potential [2,3,4]. Among these advancements, nanomaterials, with their unique physical and chemical properties, are continuously reshaping modern materials science [5]. For instance, nanoparticles (NPs), due to their large specific surface area and high reaction rates, have exhibited immense potential in fields such as biosensing, energy storage, and drug delivery [6,7,8]. However, fully understanding the exceptional properties of NPs at the atomic and molecular levels, and achieving precise atomic-scale manipulation to create advanced materials with novel properties and enhanced functionality, remains a significant challenge. Overcoming these challenges will be a decisive step toward unlocking the full potential of nanomaterials.

**Figure 1:**
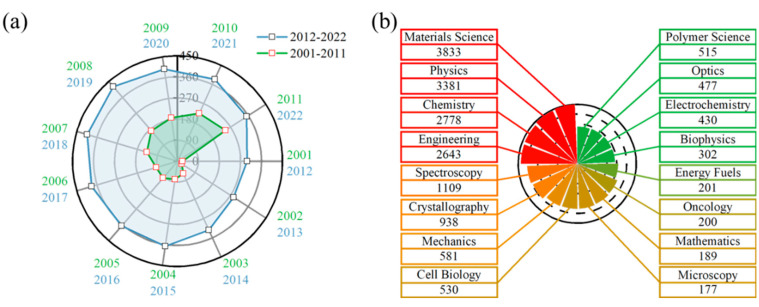
Evolution of interest in ND research: (**a**) Annual growth rate of publications related to “ND”. (**b**) Total number of publications on “ND” across various scientific disciplines.

Nanodiamonds (NDs) represent one of the most representative research systems in the field of nanomaterials. Since NDs were first reported in 1963, it has been observed that their unique extended C-sp3 structure has endowed them with exceptional mechanical, thermal, and optical properties, as well as a high specific surface area and tunable surface structure. These characteristics have led to significant advancements in battery materials, catalysts, and high-heat-density devices [9,10,11]. For instance, Liu et al. developed a multifunctional ND inorganic protective layer on zinc and copper electrodes, effectively inhibiting dendrite growth and zinc anode corrosion, thereby enhancing the cycling stability of zinc metal anodes [12]. Similarly, Shao et al. employed an annealing strategy to regulate the ketonic carbonyl content on ND surfaces, activating peroxymonosulfate to generate singlet oxygen, which efficiently oxidized organic pollutants and improved the oxidative efficiency of the catalyst as well as water remediation performance [13]. Additionally, Matsubara et al. further investigated the thermal conductivity of ND particles and their structural dependence through molecular dynamics (MD) simulations, confirming their superior thermal performance [14]. NDs exhibit exceptional mechanical and optical properties, high specific surface area, and tunable surface structures, indicating their vast potential for applications across multiple domains. Figure 1 illustrates the annual publication growth related to NDs and the total number of publications across various fields. Since the 21st century, NDs have attracted considerable research interest; despite facing numerous challenges and bottlenecks, researchers continue to actively seek solutions to advance this field. During the preparation of this article, a comprehensive analysis was conducted on the research findings related to NDs across various academic disciplines over the past 22 years. According to preliminary statistics, the total academic publications on related topics have surpassed 18,000. It is important to note that this statistic does not include the literature published under similar topics or the related papers that may have limited publication counts due to smaller research scopes. Nevertheless, as the applications of NDs continue to expand, achieving atomic-level control of these nanomaterials remains a critical challenge.

To achieve atomic-level control of nanomaterials, scientists have proposed several effective strategies that can generally be categorized into two main approaches. The first approach focuses on utilizing advanced instruments to explore the fine structures of nanomaterials in greater depth [15,16]. For example, Martín-Sánchez et al. employed optical extinction spectroscopy and small-angle X-ray scattering to reveal the structural evolution of NPs under pressure in real time [17]. The second approach emphasizes the application of advanced algorithms to analyze the structural evolution of nanomaterials [18]. For instance, Pan et al. used density functional theory (DFT) calculations to investigate the structures, mechanical properties, and electronic characteristics of several typical nanoclusters [19]. However, due to preparation challenges and other factors, relying solely on experimental techniques to decipher the fine structure of nanomaterials often causes high costs and issues with signal ambiguity. At the same time, the application of DFT methods at the nanoscale also presents limitations and fails to fully meet the demands of practical research [20]. Therefore, a comprehensive approach that integrates reported fine structural data with precise MD simulations is expected to provide an effective structural foundation for the precise manipulation of nanomaterials.

Given the exceptional physicochemical properties of NDs and the urgent need to systematically decode the fine structures of nanomaterials, we combined DFT calculations, lattice dynamics (LD), and MD techniques to explore the size effects, core–shell structures, and surface amorphization control of ND particles at the atomic level. Initially, we employed DFT calculations, incorporating finite-size phase transition effects to rigorously validate the accuracy of the Tersoff potential in describing interparticle interactions [21]. Subsequently, LD and MD methods were utilized to investigate the size effects of ND particles, and a core–shell model was constructed to explore the mechanism of surface amorphization [22,23]. Finally, using density functional theory software, we compared our simulation results with data from related literature to verify the validity and reliability of our approach [24]. This study opens new avenues for the diversified application of NDs, thereby advancing the development of novel materials.

## 2. Calculation Method

DFT calculations were performed using the Cambridge Sequential Total Energy Package (CASTEP) to optimize stable configurations and investigate the inherent properties of diamonds [25]. The project augmented wave method was used to treat the electron-ion interactions [26,27]. The exchange-correlation interactions were handled with the generalized gradient approximation (GGA) in Perdew–Burke–Ernzerhof (PBE) and Perdew–Wang 91 (PW91) [28,29]. The van der Waals interactions were added by the DFT-D3 approach [30]. After achieving the required structural accuracy, phonon dispersion curves and phonon density of states were calculated using a PBE exchange-correlation potential with a 4 × 4 × 4 k-point grid [31]. The energy cutoff was set to 400 eV, and the convergence criteria for energy and forces during full structural relaxation were established at 1 × 10^−5^ eV per atom and 0.03 eV/Å, respectively. LD simulations were carried out using the General Utility Lattice Program (GULP, version 6.1.2) [32], employing Tersoff [33,34], Brenner [35], and EDIP-Marks [36] interatomic potentials. Simulations were conducted under constant-pressure and constant-temperature (NPT) conditions for 100 ps at a temperature of 0 K and pressure of 0 GPa. Simulating under ground-state conditions (0 K and 0 GPa) is a widely accepted standard practice in the academic community, and it has been proven to be an essential approach for investigating the fundamental properties of materials [37]. This setup aims to provide an idealized reference state, simplifying physical phenomena in more complex environments, and offering a clearer analytical framework for a deeper understanding of the material’s fundamental behaviors [38]. X-ray diffraction (XRD) data for the crystal structure were analyzed using the Reflex module in Materials Studio 2020. Young’s modulus (Y), shear modulus (G), Poisson’s ratio (v), and bulk modulus (K) were calculated using the strain-stress method based on the Voigt-Reuss-Hill average scheme [39].

In comparison to DFT calculations, MD methods demonstrate better applicability in capturing the dynamic evolution of crystal structures under realistic conditions, particularly in studies involving constrained conditions or large-scale systems [40,41]. In this study, we first constructed an ideal nanocrystal based on the unit cell of a diamond and extracted appropriately sized ND particles [42]. GULP was used to investigate the thermal stability and size effects of different morphologies of ND particles at various sizes [32]. Simulations were conducted at 0 K using the Tersoff potential under NPT ensemble conditions [43]. Furthermore, to better understand the core-shell structure of the ND particles, typical MD calculations were performed using the Large-scale Atomic/Molecular Massively Parallel Simulator (LAMMPS) software (version 29 October 2020) package [44]. By referencing spherical ND particles, we examined the core–shell structure in depth by calculating radial distribution functions and coordination numbers for different sizes. Additionally, our amorphous structures were generated using the standard Tersoff potential [33,34] via a melt-quench technique. All MD simulations were executed with the LAMMPS package, while GULP was utilized for structural optimization [41]. We first constructed the initial models using LAMMPS and the Amorphous Cell (AC) module in Materials Studio 2020, respectively, based on the packing coefficient. High-temperature relaxation at 7000 K was performed until the atoms lost memory of their initial positions, fully melting the structure, followed by rapid quenching for 188 ps. This ensured the removal of metastable states, as indicated by the potential energy of the system remaining unchanged with annealing time [45]. Subsequently, the system was equilibrated in the constant-volume and constant-temperature ensemble [46,47] at 300 K for a total of 500 ps, maintaining constant particle number, volume, and temperature. Finally, one-dimensional and two-dimensional number density calculations for the optimized spherical ND particles were performed using Density Calculator (a flexible and user-friendly code for computing densities in VMD) [24]. The detailed LD and MD simulation calculation procedures can be found in the Supporting Information S1–S3.

## 3. Results and Discussion

### 3.1. Structural Characterization of NDs

**Figure 2:**
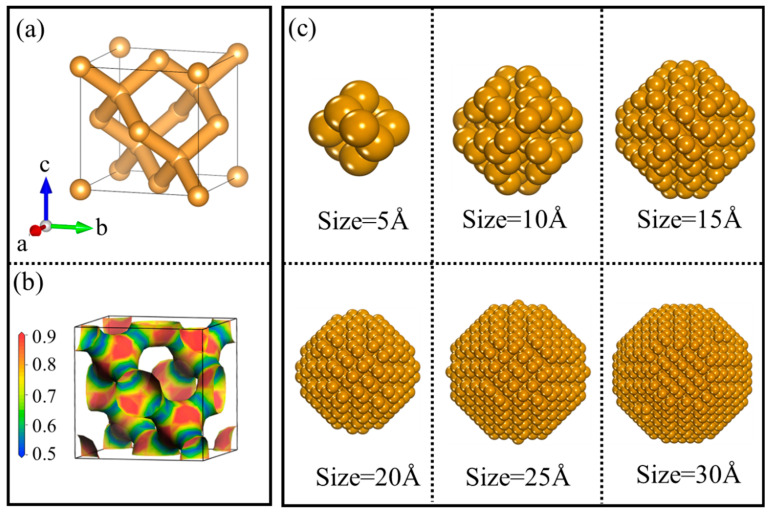
Crystal structure, charge distribution, and particle models of diamond: (**a**) Schematic of the crystal structure of NDs. (**b**) Charge distribution of NDs. (**c**) Models of ND particles with diameters of 5 Å, 10 Å, 15 Å, 20 Å, 25 Å, and 30 Å.

Before conducting an in-depth study of the surface structure of NDs, it is essential to comprehensively understand the crystal structure of diamonds. The unique crystallographic features of diamonds impart exceptional hardness, outstanding corrosion resistance, and excellent compressive strength, showcasing its immense potential in high-performance material applications. As shown in Figure 2a, the crystal structure of diamonds consists of orthotetrahedra formed by covalent bonds. Within this protocell structure, two kinds of unequal atoms are located at (0, 0, 0) and (1/4, 1/4, 1/4). Additionally, diamonds exhibit m-3m symmetry (space group Fd-3m) and belong to the face-centered cubic lattice (a = 3.567 Å, b = 3.567 Å, c = 3.567 Å), with each crystalline cell containing eight carbon atoms, which is consistent with other findings. Each carbon atom’s four valence electrons hybridize in an sp^3^ manner to form four equivalent atomic orbitals, establishing covalent bonds with the four nearest carbon atoms. The C-C bond length is 1.54 Å, the bond angle is 109°28′, and the bond energy is 347.5 kJ/mol. When using the center of each C-C bond as a point of symmetry, the six carbon atoms connected to the ends of the C-C bonds form a staggered arrangement, resulting in a highly stable conformation. According to previous studies, the chemical stability and wear resistance of diamonds are attributed not only to the staggered arrangement of carbon atoms but also to the strong covalent bonding within the diamond structure [48]. As shown in Figure 2b, a significant accumulation of charge exists between the carbon atoms in diamonds, exhibiting a spherical distribution characteristic of covalent bonding. It is well known that a diamond’s stable crystal structure and charge distribution contribute to its outstanding physicochemical properties, suggesting that under clustered conditions, the overall performance of ND particles may be even more remarkable. In summary, diamonds exhibit superior characteristics in terms of crystal structure, bond energy, and charge distribution, resulting in enhanced stability and broader applicability.

It is important to note that some scholars claim that when particle sizes decrease to the range of 1–2 nm, i.e., at the cluster scale, nearly all atoms reside on the surface [49]. As shown in Figure 2c, under the influence of a high proportion of surface atoms, significant lattice distortion occurs in ND particles. Typically, we consider the surface of NPs as a continuous sphere; however, regarding their local structure, the entire surface of the NPs is composed of numerous small crystallographic facets. Due to the high specific surface area, NDs exhibit poor thermal stability. Logically, this is disheartening news. Nevertheless, another compelling study indicates that the high proportion of active surface significantly alters the thermodynamic properties of NPs compared to their bulk counterparts, exhibiting notable size effects [50]. Additionally, the instability of ND particles causes the system to spontaneously reduce its surface area to lower surface energy, thereby decreasing the total energy of the system. However, this contraction of particles increases the distortion energy of the system, which in turn raises the overall energy. Yet, when the surface morphology of ND particles achieves a state of opposing unity with lattice distortion, meaning the system reaches a stable state, the total energy of the system is minimized. Therefore, we will next explore the relationship between the surface morphology of NDs and particle structure to further guide the development, design, and application of nanomaterials.

### 3.2. Selection and Validation of Diamond Interatomic Potential

Research indicates that the surface morphology and particle structure of ND exhibit distinct size dependencies [51]. However, current research on NDs is still inadequate, which limits their potential applications in the field of surface chemistry multifunctionality. This is primarily because analyzing the size effects of NDs presents an unprecedented challenge. The present study utilizes MD and LD simulations, which require the integration of multiple dimensions from atomic to macroscopic scales. Furthermore, due to the high number of atoms in ND clusters, DFT calculations are inefficient and computationally intensive when dealing with large systems, rendering them unsuitable. In this context, it is necessary to adopt accurate interatomic potential for simulation analysis to effectively study and predict the properties and behaviors of NDs. Next, the evaluation and selection of appropriate interatomic potential will be detailed through four aspects: E-V curves, XRD, phonon properties, and mechanical properties.

**Figure 3:**
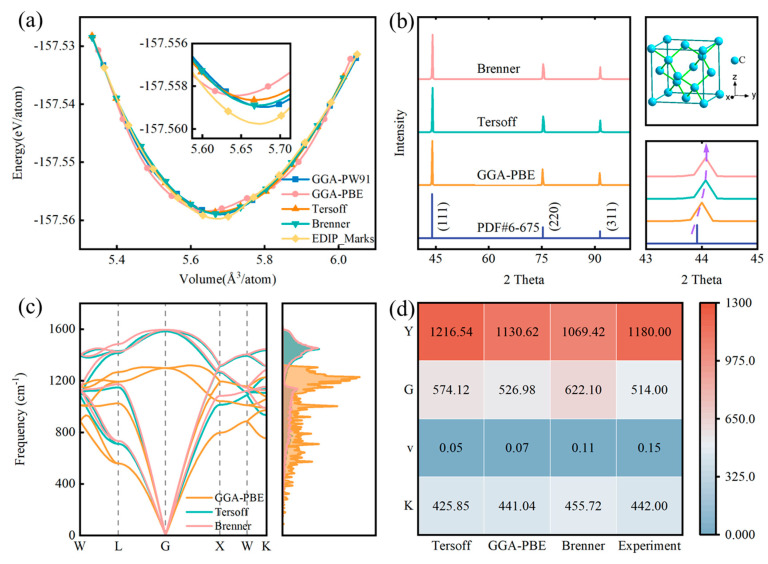
Validation of the interatomic potential of diamond: (**a**) E–V curve. (**b**) XRD spectrum. (**c**) Phonon dispersion curves and phonon density of states. (**d**) Mechanical properties.

To select the appropriate interatomic potential, we calculated the E-V curves of the diamond crystal structure using different interatomic potentials and pseudopotentials to evaluate their ability to reproduce DFT energies. Figure 3a compares the E-V curves of three interatomic potentials and two pseudopotentials: GGA-PW91, GGA-PBE, Tersoff, Brenner, and EDIP_MARKS. It is evident that the energy calculated for diamonds at the same volume using the LD method closely matches the DFT results. Notably, in the GGA-PBE results, the energy of a diamond at the equilibrium volume is −157.5582 eV/atom, while the energy from the Tersoff potential is −157.5586 eV/atom. For GGA-PW91, Brenner, and EDIP_MARKS, the energy differences at the equilibrium volume are higher than those from Tersoff and significantly deviate from the GGA-PBE results. In addition, the lattice constant at equilibrium volume is in close agreement with the experimental measurement of 3.56694 Å measured by D. P. Riley [52]. Therefore, it can be concluded that the Tersoff potential is suitable for modeling the covalent bonding environment of a diamond.

After thoroughly examining the effectiveness of the Tersoff potential in simulating the covalent bonding environment of diamonds, we further validated the accuracy and applicability of the diamond crystal structure through XRD patterns. Figure 3a presents the room-temperature XRD curves of diamonds optimized using the Tersoff potential, Brenner potential, and GGA-PBE pseudopotential. In all optimized structures, Bragg diffraction peaks corresponding to the (111), (220), and (311) crystal planes can be observed at various 2θ angles. As shown in Figure 3b, all Bragg reflections can be indexed and identified based on the face-centered cubic structure (space group Fd-3m), where carbon atoms are located at (0, 0, 0) and (1/4, 1/4, 1/4). Furthermore, no detectable impurity peaks are found in any of the optimized structures, indicating that the optimized structure remains a high-purity single-phase material. From the peak positions corresponding to the aforementioned indices, the lattice constants a can be extracted, yielding values of 3.5657 Å, 3.5656 Å, and 3.5694 Å for the Brenner, Tersoff, and GGA-PBE potentials, respectively, which are consistent with the equilibrium volume obtained from the E-V curves (Figure 3a). The differences in lattice constants between the Brenner and Tersoff potentials are minimal. As shown in the enlarged XRD curves in Figure 3b, the differences in the (111) reflection angles for diamonds optimized with the Tersoff potential, Brenner potential, and GGA-PBE pseudopotential are negligible. However, for NDs, the Tersoff potential is widely used because it effectively simulates the carbon–carbon interactions in diamonds and adequately accounts for the characteristics of the crystal structure [53].

It is well known that the ability to describe LD is crucial for evaluating the performance of interatomic potential models [54]. Therefore, to further assess the accuracy and reliability of the Tersoff potential, we calculated the phonon dispersion curves and phonon density of states for diamonds, which describe atomic vibrations. As shown in Figure 3c, the phonon dispersion curves computed using the Tersoff potential are directly compared with the results obtained from the GGA-PBE and Brenner classical potentials. The overall performance of the Tersoff potential is quite satisfactory, aligning closely with the DFT results. Specifically, the Tersoff potential accurately predicts both the low-frequency and high-frequency branches. It is noteworthy that some discrepancies occur near the gamma point, possibly due to the limitations of classical potential models, which may not accurately describe material systems involving strong electron-phonon interactions or high-temperature effects. In contrast, the performance of the Brenner potential is unsatisfactory, particularly concerning the high-frequency optical phonon branches. The significant overestimation of the high-frequency phonon branches by the Brenner potential is also evident in the density of states.

In LD simulations, the selected interatomic potential determines the forces acting on atoms and their motion behavior within the system. Accurately selecting and applying the interatomic potential is crucial for obtaining precise atomic trajectories. To validate the correctness and accuracy of the interatomic potential, we reproduced the physical and mechanical parameters of diamonds. As shown in Figure 3d, we assessed the mechanical properties of diamond structures optimized using the Tersoff and Brenner potentials, including Y, G, v, and K, and compared the specific values with the DFT and experimental results [55]. The results indicate that the mechanical properties predicted by both the Tersoff and Brenner potentials align well with DFT, with the Tersoff potential exhibiting higher accuracy. The lower accuracy of the mechanical properties predicted by the Brenner potential may be related to the significant overestimation of the high-frequency phonon branches in diamonds, as shown in Figure 3c. Thus, it is feasible to solve Newton’s equations using the Tersoff potential to calculate the position and momentum of particles at any given moment, thereby revealing the evolution of the system’s organization and performance. Overall, while DFT calculations based on electronic scales provide high precision, they are computationally intensive. In contrast, classical MD methods simplify the description of interatomic interactions using empirical or semi-empirical potentials and simulate atomic motion based on Newtonian mechanics, effectively reducing computational demands and making it feasible to simulate the performance of large systems at the atomic scale. Based on crystallography (E-V curves, XRD), LD (phonon dispersion curves, phonon density of states), and mechanical properties (Y, G, v, K), along with related experimental reports, it is confirmed that the Tersoff potential can accurately describe interatomic interactions, facilitating its application in the study of size effects in NDs.

### 3.3. Fine Structure Anatomy

In the study of NDs, the Tersoff potential provides a reliable method for describing their size effects and surface structures. However, as the actual environment changes, the morphology and structure of NDs undergo dynamic adjustments, necessitating more precise particle models to enhance the alignment between simulations and experiments. Therefore, we will focus on surface morphology, lattice distortions, and particle energy to more accurately describe the properties of NDs. Research indicates that spherical NDs exhibit superior performance in terms of size effects and stability. Furthermore, we will develop a more detailed spherical particle model from the perspectives of atomic positions (RDF) and stability (coordination number) to gain deeper insights into their structural features.

#### 3.3.1. Size Effect of Diamond NPs

**Figure 4:**
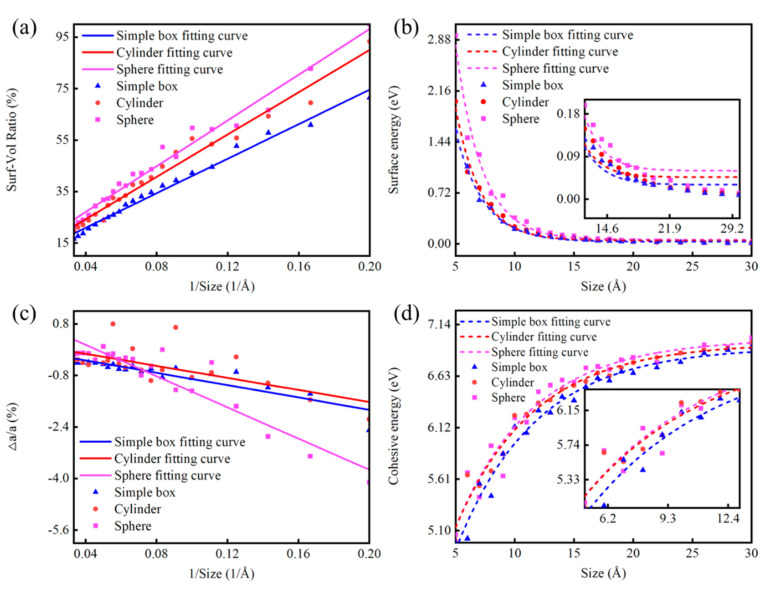
Size effects of NDs with diameters ranging from 5 to 30 Å: (**a**) Surface atom proportion. (**b**) Surface energy. (**c**) Strain rate. (**d**) Cohesive energy.

It is well known that as particle size decreases to the nanoscale, the surface area-to-volume ratio significantly increases [56]. At the nanoscale, many properties, such as catalytic activity and optical characteristics, undergo notable changes due to the high proportion of surface atoms [42]. To assess the surface atom proportion of the ND particles, we manually counted the atoms exposed on the surface. Figure 4a illustrates the proportion of surface atoms for NDs of varying sizes and shapes, calculated using the Tersoff potential. All these ND particles exhibit a similar trend: the proportion of surface atoms increases as the particle size decreases. We also observed that, within the nanoscale range, particle shape plays a crucial role: at the same size, spherical particles have the highest proportion of surface atoms, while simple cubic particles have the lowest. Furthermore, within the range of 5 to 20 Å, the size and shape of the particles have a significant impact on their properties. As the particle size increases to 25 to 30 Å, the proportion of surface atoms gradually approaches 20%, which indicates that the surface atom ratio stabilizes and is gradually no longer significantly affected by changes in particle size or shape.

The energy size effect in NPs is primarily driven by the changes in their electronic structure and quantum confinement as the particle size decreases [57]. Surface energy is another crucial surface property that directly affects the physical performance of materials, including elasticity, thermal stability, diffusion, and strength [58]. Figure 4b illustrates the trend of surface energy per atom for ND particles as a function of size. The curves in Figure 4b clearly indicate the following: (a) Surface energy increases as particle size decreases; (b) Surface energy decreases with increasing particle size, with a more pronounced trend for particle diameters less than 10 Å compared to those greater than 10 Å. These observations suggest that for smaller particle sizes (∼<10 Å), the contribution of excess energy from the surface is significant, whereas this contribution markedly decreases as particle size increases (exceeding 10 Å). Additionally, the surface energies of NDs with the same size but different shapes also vary. The surface energy is minimal for simple cubic structures, followed by cylindrical structures, and is highest for spherical structures. According to our findings, the effect of size on the surface energy of spherical ND particles is particularly dominant in the range of approximately 0.5 to 2.0 nm.

The non-uniform distortion of the lattice is critically important in the study of surface properties of conventional crystalline materials, and this non-uniform lattice distortion is even more pronounced for NPs at the nanoscale [59]. To assess the distortion rate of ND particles, we employed the edge atom coordinate subtraction method to calculate the maximum vertical interatomic distances in the x, y, and z directions. Based on the average values obtained for these maximum vertical interatomic distances, we calculated the distortion rate of ND particles using the formula $\alpha =\frac{∆a}{a}$. The distortion rates for NDs of different shapes, as a function of particle size, are shown in Figure 4c. The trend indicates that the distortion rate increases as particle size decreases. This result is consistent with the findings of Yu et al. [60], who reported that the lattices of Sn and Bi NPs contract with decreasing particle size. As the particle size approaches the nanoscale, the lattice periodically terminates, leading to a reduction in the coordination number (CN) of surface atoms [61]. Smaller particle sizes correspond to lower coordination numbers. The imperfection in atomic coordination results in the contraction of residual bonds for low-coordination atoms. Therefore, for NPs free of impurities, the reduction in coordination typically leads to shorter bond lengths at the surface, ultimately causing lattice contraction.

To compare the relative stability of NDs, we calculated the cohesive energy per carbon atom for NDs of different sizes and shapes. We defined the cohesive energy per carbon atom using the formula $\Delta E=\left[E_{tot(Cn)}-nE_{C}\right]/n$, where *E_tot_*_(*Cn*)_ is the total energy of the ND, *E_C_* is the energy of a single carbon atom, and n is the number of carbon atoms in the ND. Figure 4d displays the calculated cohesive energy per atom for NDs of various sizes and shapes. The results indicate that as the size of the NPs increases from 0.5 nm to 3 nm, the absolute value of the cohesive energy per carbon atom gradually increases. Due to the presence of surface energy, the cohesive energy of surface atoms in the ND is lower than that of internal atoms, resulting in a cohesive energy that is less than that of the bulk material. As the size of diameter decreases, the ratio of surface atoms increases, leading to a reduction in cohesive energy. As shown in Figure 4d, the cohesive energy values for NPs of the same size but different shapes vary: the spherical structure exhibits the highest cohesive energy, followed by the cylindrical structure, and finally, the simple cubic structure. This variation in size and shape indicates that surface energy plays a significant role in the size stability of NDs, with larger spherical NDs displaying the most stable structure.

#### 3.3.2. Layer-by-Layer Dissection of ND Particles

**Figure 5:**
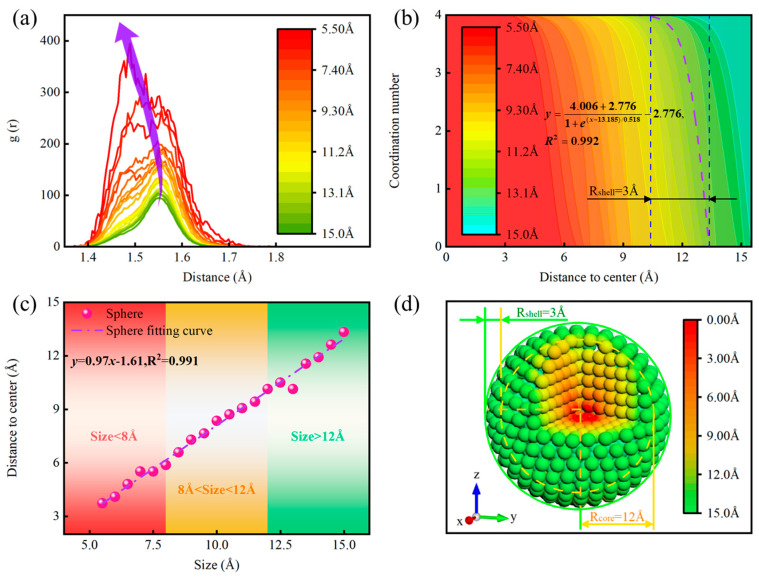
Construction of the core–shell model for NDs: (**a**) Radial distribution function for NDs with a cutoff radius of 2 Å. (**b**) Relationship between coordination number and size, where Rshell is the radius of the shell. (**c**) Relationship between the size and the coordination number’s critical point. (**d**) Core–shell structure model for an ND particle with a diameter of 30 Å, where Rcore and Rshell are the radius of the core and shell, respectively.

It is well-known that the radial distribution function (RDF) is an effective tool for analyzing the microstructure of NPs. The choice of a spherical shape is not only due to the aforementioned analysis of size effects but also because this shape is the most common in gas-phase synthesis, where rapid solidification occurs, making it widely adopted in MD simulations. We calculated the radial distribution function (RDF) for diamond clusters of twenty different sizes over a simulation time of 10 ps, as shown in Figure 5a. The figure reveals that smaller clusters exhibit higher RDF peak values compared to larger clusters. In other words, the probability of finding cluster atoms around a reference atom is greater in smaller clusters than in larger ones. This may be attributed to the more pronounced structural distortion of small atomic clusters compared to their larger counterparts. This observation is supported by the snapshots of diamond nanoclusters of different sizes presented in Figure 2c. Furthermore, for ND particles with a radius below 7 Å, the second peak is notably split compared to other sizes, which is related to the presence of locally ordered icosahedral structures within the system [62]. Additionally, Figure 5a indicates that as the size of the ND particles decreases, the RDF peaks shift to the left (i.e., toward smaller angles). This suggests that the reduction in the size of ND particles enhances C/C interactions. Moreover, the RDF peaks for small-sized amorphous ND particles are broader than those for larger sizes, indicating that the former possesses a more heterogeneous structure compared to the latter, likely due to contributions from the surface structure, similar to what is typically observed in amorphous solid metals.

The evidence from the aforementioned studies indicates that the size effect and atomic distribution of ND particles depend on both particle size and crystal structure; however, the exploration of shell structures that can achieve satisfactory catalytic properties remains an area for further investigation. The relationship between the coordination number of ND particles of varying sizes along the radial direction is illustrated in Figure 5b. The fitting equation for the coordination number of ND particles with a radius of 13 Å as a function of center distance is presented as follows:

(1) $$y=\frac{4.006+2.776}{1+e^{(x-13.185)/0.518}}-2.776,R^{2}=0.992$$

In this context, *y* and *x* represent the coordination number and center distance of the ND, respectively. The coefficients in the equation have the following interpretations: 4.006 represents the asymptotic value of the function at the upper limit (or as *x* → −∞), −2.776 represents the asymptotic value at the lower limit (or as *x* → +∞), 13.185 corresponds to the x-value at the inflection point where *y* = 0.615, and 0.518 denotes the slope of the curve at this inflection point. As shown in Figure 5b, the coordination number of particles with radius ranging from 5.5 Å to 15 Å decreases stepwise at approximately 3 Å from the surface layer (with a coordination number of 0). This finding indicates that the surface activity of dehydrogenated NDs arises from the unsaturation of the coordination number of surface atoms in the surface layer, while the surface of real NDs is typically terminated by hydrogen atoms, which can be clearly observed in NMR measurements [63]. Consequently, this model can be employed to simplify the representation of NPs. DFT calculations are utilized to evaluate the shell layer, allowing for a more nuanced understanding of the electronic properties, while calculations for the internal core are performed using MD. The combined approach of DFT and MD has been successfully applied in materials science and computational chemistry, such as the QM/MM method. This method integrates the accuracy of quantum mechanics with the efficiency of molecular mechanics by partitioning the system into quantum and molecular mechanical regions, thereby achieving a balance between precision and efficiency [64,65]. The multiscale simulation holds promise for enabling a more rational and efficient design of high-performance ND catalysts.

Understanding the core–shell structural partitioning of ND particles is indeed a challenge, particularly since relying solely on the relationship between coordination number and center distance may not provide sufficient quantitative information. Figure 5c illustrates the relationship between the transition points of the coordination number and the sizes of ND particles, thereby clarifying the connection between particle size and changes in coordination number. The fitting equation is presented as follows:

(2) $$y=0.97x-1.61,R^{2}=0.991$$

In this context, *y* and *x* represent the transition points of the coordination number corresponding to the center distance and size of the NDs, respectively. The results indicate that, within the nanoscale range, as the particle size increases, the center distance corresponding to the transition point is proportional to the size, suggesting that the shell thickness of the nanocrystals remains relatively constant. Therefore, we can extend the core–shell model to accommodate particles of varying sizes. In this model, the internal atomic arrangement of the particles exhibits a proportional change with size, meaning that as the particle size increases, the range of ordered arrangement of internal atoms correspondingly expands, while the range of surface atoms remains largely unchanged. It is worth noting that, within the simulation error range, and when the particle size approaches 13 Å, a local decrease in the distance from the central value occurs. This is attributed to the balancing of the ratio between surface and interior atoms, resulting in a gradual weakening of surface effects. At this stage, the particle may undergo local structural optimization, with surface atoms gradually rearranging to attain a more stable state. Overall, in spherical NPs, the differing surrounding environments of surface atoms compared to internal atoms lead to abrupt changes in the coordination number of surface atoms. This variation in coordination number significantly influences the physicochemical properties of the NPs, highlighting the unique characteristics of materials at the nanoscale.

Analyzing solely from the perspectives of RDF and coordination number may prove insufficient for understanding the underlying structure. Therefore, by analyzing these data, we extract important information regarding the structure of the NPs and transform it into more specific and visualizable structural models. This approach aids in our understanding of the internal composition and arrangement of NPs. To establish a core–shell model corresponding to the MD-simulated spherical grains, we constructed an atomic model with the same number of atoms as in the corresponding MD model. The lattice model of a spherical ND particle with a radius of 15 Å is illustrated in Figure 5d. The color of the particles is coded according to the value of their distance from the center, with particles close to the core being red, indicating a shorter distance; as the distance increases, the color gradually transitions to yellow and finally to green at the shell. From the structural schematic, it is evident that the nanoparticle’s core–shell structure is heterogeneous, consisting of two distinct components: a central core and an external shell. In the structural diagram, the core typically represents the central region, while the shell surrounds the core. Despite variations in the overall particle size, the shell thickness remains relatively stable, typically on the order of approximately 3 Å. This structural design enables the NPs to achieve tunable electronic, optical, or chemical properties between the core and shell. For instance, the core may provide the primary functional characteristics, while the shell can enhance stability, modulate reactivity, or introduce surface functionalization, thus exhibiting superior performance in fields such as catalysis, drug delivery, or sensors [66].

#### 3.3.3. Surface Amorphous Structure Modulation

It is well established that the specific surface energy of amorphous solid metals is greater than that of amorphous liquid metals but lower than that of crystalline metals. This leads to a tendency for crystalline metal surfaces to transition to an amorphous state. However, the structural definition of amorphous materials is relatively vague, resulting in a broad range of values for the first coordination number (kn) and packing coefficient (kp) traditionally used to characterize amorphous structures. At the nanoscale, the surface atoms of diamond particles, being exposed to the external environment, typically no longer form a perfect crystalline structure with the internal atoms. Compared to the internal atoms, these surface atoms have a lower coordination number, making them more prone to local disorder and the formation of an amorphous surface structure. In this study, the amorphous structure of the nanodiamond surface is defined by adjusting the system volume to achieve the desired packing coefficient, with the kn(kp) relationship analyzed to determine the kn(kp) function for different packing coefficients ranging from 0.30 to 0.48. The study found that the correlation of kn(kp) could be described by the following quartic polynomial, regardless of whether LAMMPS modeling followed by quenching and GULP structural optimization or AC modeling followed by LAMMPS quenching and GULP structural optimization was used:

(3) $$y=110.8-1162.0x+4636.9x^{2}-8089.3x^{3}+5224.2x^{4},R^{2}=0.99$$

**Figure 6:**
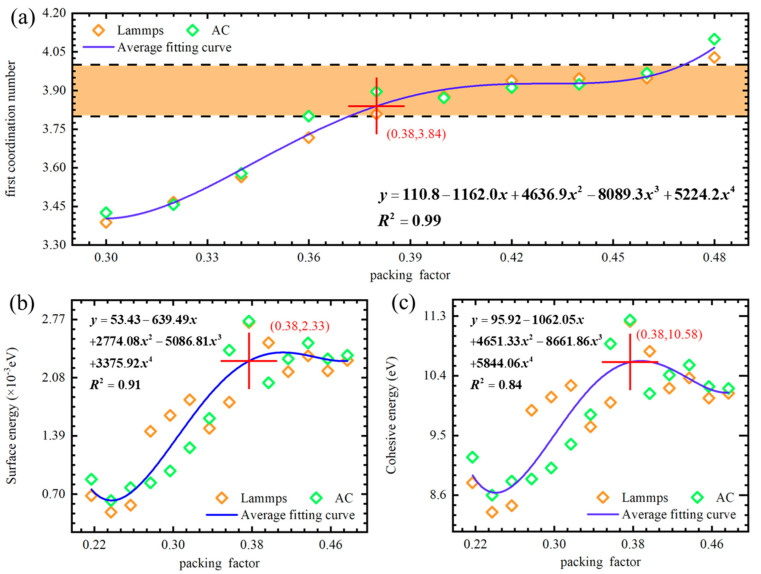
In-depth study of the amorphous structure: (**a**) Correlation between packing coefficient and first coordination number for NDs. (**b**) Relationship between packing coefficient and surface energy. (**c**) Analysis of the relationship between packing coefficient and cohesive energy.

In this context, *y* and *x* represent the first coordination number and packing coefficient of the amorphous structure of nanodiamonds, respectively. As illustrated in Figure 6a, this dependency exhibits an N-shaped curve, indicating that within the range of 3.852 ≤ kn ≤ 3.958, the kn value remains relatively constant with respect to the kp value, which ranges from 0.38 to 0.46. Consequently, the corresponding region has been referred to as the “random packing” region, where compressive and tensile strains are present throughout the amorphous structure [67]. It can be inferred that the plateau region of the kn(kp) curve corresponds to the amorphous structure of nanodiamonds, specifically within the ranges of 0.38 ≤ kp (amorph) ≤ 0.46 and 3.852 ≤ kn (amorph) ≤ 3.958.

To validate this hypothesis, it is necessary to calculate the properties of amorphous metals. Due to the lack of long-range order, the surface energy of amorphous materials is typically higher. This higher surface energy can result in increased reactivity, affecting the stability and chemical behavior of the material. The relationship between the surface energy of the amorphous ND structure (in units of 10^−3^ eV) and the packing coefficient is shown in Figure 6b. The orange symbols represent results obtained from Lammps modeling followed by quenching and GULP structural optimization. The green symbols represent results from AC modeling combined with Lammps quenching and GULP optimization. The red crosses indicate the point where the surface energy change begins to level off. The blue solid line represents the average surface energy obtained from both Lammps and AC modeling. As shown in Figure 6b, the surface energy exhibits a plateau across different modeling approaches, leveling off at a packing coefficient of 0.38. The corresponding fitted equation is as follows:

(4) $$y=53.43-639.49x+2774.08x^{2}-5086.81x^{3}+3375.92x^{4},R^{2}=0.91$$

Here, *y* and *x* represent the surface energy of the amorphous structure of the ND surface and the packing coefficient, respectively. It can also be observed that although the surface energy fluctuates for packing coefficients greater than 0.38, it gradually levels off. This indicates that the atomic arrangement and interactions in the amorphous structure of the ND surface for *x* > 0.38 do not undergo significant changes under these conditions. In summary, the stability of the surface energy in amorphous materials suggests that their surface structure and chemical environment remain relatively stable under varying conditions. The surface energy is primarily determined by the local arrangement and interactions of surface atoms rather than being significantly influenced by the global packing coefficient.

To further ensure the stability of the amorphous structure on the ND surface, we also calculated the cohesive energy of this amorphous structure. Figure 6b illustrates the relationship between the cohesive energy of the amorphous ND structure and the packing coefficient. As shown in Figure 6c, the cohesive energy exhibits a plateau across different modeling approaches, leveling off at a packing coefficient of 0.38. The corresponding fitted equation is as follows:

(5) $$y=95.92-1062.05x+4651.33x^{2}-8661.86x^{3}+5844.06x^{4},R^{2}=0.84$$

Here, *y* and *x* represent the cohesive energy of the amorphous structure on the ND surface and the packing coefficient, respectively. The results show that within the packing coefficient range of 0.40 to 0.46, the cohesive energy exhibits a downward trend. However, beyond this “random packing” region, the cohesive energy begins to increase, likely due to fluctuations caused by uneven stress distribution. This stress may result from external loads or mismatches in phase transitions and thermal expansion within the material. Overall, the trend of the cohesive energy is largely consistent with that of the first coordination number and surface energy, with a significant deceleration in growth after the packing coefficient reaches 0.38. This suggests that atomic clusters with a packing coefficient greater than 0.38 form a thermodynamically stable structure. The calculations of surface energy and cohesive energy for the amorphous surface of NDs provide insights into the stability of both the surface and the core, enabling precise predictions of the material’s actual performance and potential applications. Therefore, in NPs calculations, it is often sufficient to use DFT methods to obtain the fine electronic properties of the shell, while the core can be analyzed using MD.

### 3.4. Comparative Verification

**Figure 7:**
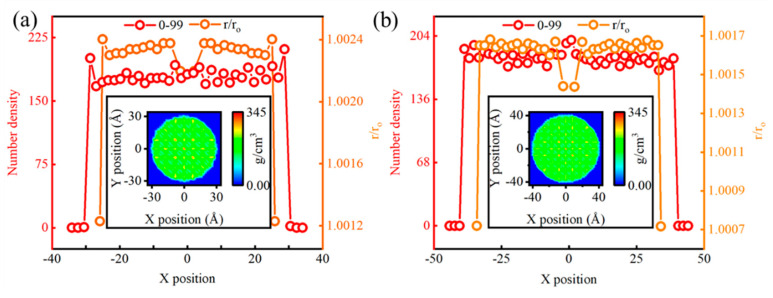
Validation of simulation results: (**a**) Number density distribution for NDs with a radius of 60 Å. (**b**) Number density distribution for NDs with a radius of 80 Å.

Relying solely on the comparison between DFT calculations and empirical interatomic potential to justify the accuracy of our simulations may not be sufficiently convincing. Therefore, it is necessary to further validate our results by comparing them with those reported in the relevant literature. Given that material properties are dictated by structural configurations, we approach this analysis from the perspective of local atomic structure distortions. Figure 7 presents 1D number density distributions and the variation of r/r_0_ with distance from the grain center, calculated by averaging 100 frames (0 to 99) for grains with diameters of 60 Å and 80 Å. The ratio r/r_0_ is consistently greater than 1, indicating that the “average” atomic lattice of NDs is expanded compared to that of a perfect diamond crystal. This result is consistent with previous studies [68], which concluded that materials tend to expand during the amorphization of the crystal structure. This lattice expansion increases as the grain size decreases: in the 1D number density plot, the number densities at r/2 for 60 Å and 80 Å ND particles are approximately 181.4 and 196.8, respectively. The variation in atomic number density observed in the MD models is attributed to the presence of local tensile and compressive strains within the grain volume. These strains exhibit symmetry corresponding to the spherical shape of the grains. These strain patterns can be further visualized through density calculation software [24]. From the 2D number density distributions averaged over 100 frames (0 to 99) for the optimized structures of 60 Å and 80 Å grains, it is evident that smaller grain sizes tend to lead to higher atomic number densities and greater surface distortions, while larger grain sizes result in a more uniform internal structure and lower surface density. The consistency of this structural behavior suggests that, despite variations in particle size and shape in different regions, the evolution of their internal structures follows similar patterns. This finding not only aligns with theoretical predictions in the literature but also further confirms the effectiveness and accuracy of our simulation approach. By demonstrating a high degree of consistency between our simulation results and those from relevant studies, we validate the accuracy of the model and methods employed. This not only attests to the reliability of our simulations but also provides a robust theoretical foundation for further exploration of the relationship between the microstructure and macroscopic properties of materials.

Although our model assumes an idealized ND surface, the actual surface is far more complex due to the presence of various functional groups and defects. NMR and EPR studies have shown that surface modifications, such as oxidation and graphitization, significantly affect the material’s properties, and these effects are often size-dependent, with surface reactivity and behavior varying based on nanoparticle size and surface structure [63]. To address this complexity, our study focuses on an idealized ND surface to provide a clearer understanding of the fundamental properties and intrinsic structure of the material rather than replicating real-world conditions. We have explicitly noted that these simulations are conducted under idealized boundary conditions to establish a theoretical framework for future research. While these findings may differ from actual environmental conditions, they remain critical for advancing the understanding of the material’s fundamental physical characteristics.

## 4. Conclusions

We conducted a comprehensive study of the size effects, core–shell structure, and surface amorphization of NDs using DFT calculations, LD, and MD simulations. The fine structural analysis revealed that the atomic arrangement within the core exhibits a proportional change with particle size—the larger the particle, the broader the range of ordered atomic arrangement in the core—while the arrangement of surface atoms remains largely unchanged. DFT calculations were employed for the shell layer to obtain more detailed electronic characteristics, while the core was analyzed using MD methods. The results of amorphous structure regulation further demonstrated that the atomic arrangement and interactions in the surface amorphous layer of NDs with a packing coefficient greater than 0.38 showed no significant changes. This study provides a solid theoretical foundation for the multiscale simulation of NDs and is expected to advance simplified calculations and atomic-level manipulation of NPs in the field of nanomaterials.

## Data Availability

The original contributions presented in this study are included in the article material, and further inquiries can be directed to the corresponding author.

## Supplementary Materials

The following supporting information can be downloaded at: https://www.mdpi.com/article/10.3390/nano14242024/s1, S1: Lattice dynamics (LD) calculations. S2: Molecular dynamics (MD) calculations of crystal structure. S3: Molecular dynamics (MD) calculations of amorphous structure.
