# Supplementary material for: Deciphering Surface-Localized Structure of Nanodiamonds
Source: Nanomaterials (Basel). 2024 Dec 16;14(24):2024. doi: 10.3390/nano14242024 (PMC11728798; doi:10.3390/nano14242024)
Supplement: Supplementary file 1 [file nanomaterials-14-02024-s001.zip › nanomaterials-3349058-supplementary.pdf]

Supporting Information

Deciphering the Surface-Localized Structure of Nanodiamonds

Li Ma[1], Zhijie He[1], Keyuan Chen[1], Hanqing Li[1], Yongzhi Wu[1], Jueyi Ye[1], Hongying Hou[1], Ju Rong[1*] and Xiaohua Yu[1,2*]

[1]Faculty of Materials Science and Engineering, Kunming University of Science and Technology, Kunming, 650093, China

[2]Yunnan Key Laboratory of Integrated Computational Materials Engineering for Advanced Light Metals, Kunming, 650093, China

[*]Correspondence: JRong_kmust@163.com; xiaohua_y@163.com

List of Contents

S1: Lattice dynamics (LD) calculations.

S2: Molecular dynamics (MD) calculations of crystal structure.

S3: Molecular dynamics (MD) calculations of amorphous structure.

S1 *Lattice dynamics (LD) calculations*

As the particle size decreases, the physical and chemical properties of the material undergo significant changes, which are crucial for the application of nanomaterials. In this study, we used the General Utility Lattice Program (GULP) to calculate the surface morphology, lattice distortion, and particle energy of nanodiamond (ND) particles of different shapes and sizes, in order to investigate the thermal stability and size effects of nanodiamonds [1]. The simulations were performed at 0 K using the Tersoff potential under constant-pressure and constant-temperature (NPT) conditions [2]. Structural optimization and static calculations were first carried out for both the initial and final states using GULP to obtain stable configurations. Finally, the distortion rate was calculated using the "atomic coordinate subtraction" method. The distortion rate was determined by the maximum variation in the distance between edge atoms along the x, y, and z directions, and the average value was obtained to determine the non-uniform distortion rate. Based on the diamond unit cell, we used Material Studio to construct ND models of various sizes and shapes. This work is a theoretical study: the models created represent pure, perfect ND particles.

S2 *Molecular dynamics (MD) calculations of crystal structure*

To better understand the core-shell structure of ND particles, molecular dynamics (MD) simulations were performed using the Large-scale Atomic/Molecular Massively Parallel Simulator (LAMMPS) software package [3]. Spherical ND particles were chosen as the reference model, and the radial distribution function and coordination number were computed for different particle sizes to investigate the core-shell structure

in detail. The simulations were carried out in the NPT ensemble, and the Tersoff potential was used to model the van der Waals interactions between carbon atoms, with a coordination number cutoff range of 2 Å [4]. The temperature was maintained at 300 K with a temperature coupling time of 1.0 ps. The pressure was set to zero compression and zero translational coupling, with a pressure coupling time of 0.2 ps. In the output, time averaging was performed, with results output every 100 steps over a total of 10,000 simulation steps. The optimized structures were further analyzed for both one-dimensional and two-dimensional number density using the DensityCalculator (a flexible and user-friendly code implemented in VMD) [5].

S3 *Molecular dynamics (MD) calculations of amorphous structure*

The amorphous structure was generated using the standard Tersoff potential [4] through the melt-quench technique. All MD simulations were performed using the LAMMPS package, while structural optimization was carried out with GULP [6]. Starting from the diamond cubic lattice with a lattice constant of 3.56 Å, carbon atoms were randomly distributed within the lattice at a predefined ratio. Periodic boundary conditions were applied in all three primary directions, and each calculation cell contained 700 atoms, with the cell volume kept constant throughout the simulations. The density differences between systems were achieved by adjusting the lattice constants. Initially, we first constructed the initial models using LAMMPS and the Amorphous Cell module in Materials Studio 2020, respectively, based on the packing coefficient. The structure was first relaxed at 7000 K until the atoms lost memory of their initial positions, ensuring complete melting. Rapid quenching was then performed,

with a quench time of 188 ps, ensuring the removal of metastable states (as evidenced by the system's potential energy, which did not change with annealing time) [7]. It is worth noting that studies have shown that cooling rates exceeding 10^{16} K/s hinder normal relaxation of the structure, leading to an excessively high sp^3 fraction in amorphous carbon. Therefore, a cooling rate of approximately 10^{11} K/s was adopted to ensure proper relaxation of the structure. The system was then equilibrated for 500 ps at 300 K under constant-volume and constant-temperature (NVT) conditions [8], keeping the number of particles, volume, and temperature constant. Following these steps and constraining the domain at the specified volume, amorphous carbon structures with mass densities ranging from 2.29 to 5.01 g cm^{-3} were obtained.

References

1. Gale, J.D. and A.L.J.M.S. Rohl, *The general utility lattice program (GULP).* **2003**. 29(5): p. 291-341.

2. Nosé, S.J.M.p., *A molecular dynamics method for simulations in the canonical ensemble.* **1984**. 52(2): p. 255-268.

3. Plimpton, S., P. Crozier, and A.J.S.n.l. Thompson, *LAMMPS-large-scale atomic/molecular massively parallel simulator.* **2007**. 18: p. 43.

4. Tersoff, J.J.P.r.B., *Modeling solid-state chemistry: Interatomic potentials for multicomponent systems.* **1989**. 39(8): p. 5566.

5. Wang, Y., et al., *Calculation of 1D and 2D densities in VMD: A flexible and easy-to-use code.* **2021**. 266: p. 108032.

6. Symianakis, E. and A.J.C.M.S. Kucernak, *Embedded atom method interatomic potentials fitted upon density functional theory calculations for the simulation of binary PtNi nanoparticles.* **2017**. 133: p. 185-193.

7. Feldman, J.L., P.B. Allen, and S.R.J.P.R.B. Bickham, *Numerical study of low-frequency vibrations in amorphous silicon.* **1999**. 59(5): p. 3551.

8. Hoover, W.G.J.P.r.A., *Canonical dynamics: Equilibrium phase-space distributions.* **1985**. 31(3): p. 1695.
